# The Treatment of Various Symptoms in Nervous Diseases

**Published:** 1909-09-25

**Authors:** James Taylor

**Affiliations:** Physician to the National Hospital, and to Moorfields Eye Hospital, and Consulting Physician to the Queen's Hospital for Children


### Hospital Clinics.

### THE TREATMENT OF VARIOUS SYMPTOMS IN NERVOUS DISEASES.

By JAMES TAYLOR, M.D., F.R.C.P.; Physician to the National Hospital, and to Moorfields Eye Hospital, and Consulting Physician to the Queen's Hospital for Children.

(Delivered at the National Hospital for the Paralysed and Epileptic, Queen Square, London, July 6, 1909.)

Gentlemen,—We have all been taught to believe that nervous diseases as a class are of great intractability; that, whilst they are interesting clinically and pathologically, yet from the point of view of treatment they do not offer a wide field of interest because of the belief which is held that these diseases are very little amenable to the measures which we may employ for their relief. In some degree that is true, especially of diseases of the nervous system proper —diseases which are characterised pathologically by degenerative changes in the elements of the nervous system. But we must recognise that there are a great many nervous diseases, so-called, which are really not—primarily at all events—nervous diseases, but are diseases of other systems, in which the symptoms are due to interference with the functions of the nervous system. Take the most familiar example of this, a case of hemiplegia. No one dreams of looking at that as anything but a nervous disease, but if we regard its origin as the rupture of a vessel, we must recognise that though the symptoms are those of interference with the nervous system, the disease is really one of the vascular system. So also with reference to another common disease, a gumma of the dura mater. Suppose a patient has gumma of the dura mater, he has symptoms which are distinctly referable to the interference which this inflammatory disease of the connective tissue exerts upon the nervous system. But the disease is not really one of the nervous system, but a disease of the connective tissue. Neither a gumma of the dura mater nor a cerebral hæmorrhage can correctly be called a disease of the nervous system; but no one fails to recognise that by far the most prominent symptoms in those two diseases are those which result from interference with the function of the nervous system.

Many nervous diseases are the result of the action of toxic substances on the nervous system. And sometimes these toxic substances are in a very active and virulent form, and sometimes in an attenuated form. Let us consider diphtheritic paralysis. That is a disease in which we know there is a toxin present which poisons the nervous system. In some cases the poison which has this effect is virulent in its action, leading rapidly to the death of the patient. In other cases the poison is not nearly so virulent: the patient may only have a slight degree of weakness of the limbs; he may have some ocular palsy, with some other symptoms of interference with the functions of the nervous system; but he may after a time completely recover. I think this is a very suggestive illustration of the way in which toxins may affect the nervous system.

The disease which is known as Landry's or acute ascending paralysis is rightly regarded as one of the most certainly fatal—and very often rapidly fatal—of all nervous diseases. But any one who has had the opportunity of watching several cases of nervous disease knows that many cases of Landry's paralysis, strictly so-called, in which the symptoms are all present, in which there is evidence of some poisoning of the nervous system, completely recover. I have seen here at least three cases in which I think there was no doubt in the minds of any one who saw them that they were strictly cases of Landry's paralysis, cases of toxic paralysis which did not end in the usual way. In certain other cases there are poisonous substances which affect the nervous system when given, so to speak, in repeated doses. A patient has an illness which is characterised by some degree of paralysis, and after a time, and under appropriate treatment, that patient may get a good deal better. But presently another dose of poison appears to be elaborated, and all the old symptoms come back, but in a somewhat stronger form; and at the end of the second attack the patient is left considerably weaker than he or she was after the first attack. And that may go on indefinitely. I am drawing no fancy picture, because that is frequently the history in cases of disseminated sclerosis. In many such cases the patient has an attack of illness characterised by profound paralysis; but after a time that may pass away so completely that the patient is seen to be scarcely paralysed at all. After a time the patient has another illness, and the symptoms return with renewed force, and after the second attack they do not disappear to anything like the same degree as after the first. And that may go on indefinitely, until after a time the patient is left completely paralysed. One knows there are cases in which, apparently, the first attack of the poisoning is the last also; because one has seen cases here in which the patient has come with a history of acute illness and all the signs and symptoms of disseminated sclerosis—nystagmus, tremor of the hands and legs, and bladder difficulty—and yet with appropriate rest and other treatment that patient has recovered, and in the course of three or four years there has been no recurrence of the symptoms. So one may claim such a case as one of disseminated sclerosis which has recovered. An unfortunate thing in reference to the poison in those cases is, first of all, that we do not know what the poison is, we do not know what determines its elaboration, and we do not know any antidote for it. So, until these things are cleared up, we shall be, to a certain extent, helpless in the face of such a disease as disseminated sclerosis. Yet the recognition of the fact that a case of disseminated sclerosis does not necessarily go on to helplessness, but may recover, enables one with judgment and experience to give a much more favourable prognosis, and one which is more likely to be correct, than one is in the habit of considering likely.

There is another class of cases, in which there seems to be a gradual deterioration of structures, due to the slow but steady effect of some poison. In tabes dorsalis, of course, we have a disease of this kind, and I do not know anything more suggestive in the way of analogy than that which Sir William Gowers formulated some years ago. He was speaking of tabes dorsalis and alcoholic peripheral neuritis, and he said that peripheral neuritis from alcohol we must look upon as a toxic disease, and that the ultimate source of the poison which caused that disease is the yeast plant; and that in tabes dorsalis we have to deal with a similar toxic disease; in this case, however, the central nervous system is at fault, and the ultimate poison in this case is almost certainly the microbe of syphilis. So that we have here two parallel instances of toxic disease; two different toxins, of course, but each toxin probably traceable ultimately to a now well-known but lowly organism, one resulting in the poisoning and degeneration of the peripheral nervous system, and the other attacking the central nervous system. But it is conceivable that in any given case the supply of such poison is limited, and cases are not uncommon in which we may say the condition is one of embryonic tabes dorsalis. We know that in many instances the Argyll-Robertson pupil is present, and nothing else. In a certain number of cases there may be lightning pains, loss of knee-jerks, and nothing else. In one case I know at the present time the patient has had Argyll-Robertson pupil 15 years without developing any other sign; and so with other cases of tabes, the poison seems to have spent itself, and no more seems to be elaborated. Its effect on the nervous system ceases, though a certain amount of damage has been done which is irremediable. And when the morbid process has ceased, by means of appropriate exercises applied to the various structures which remain, we may get considerable improvement in the condition of the patient. It is this view which justifies one in the treatment of tabes dorsalis by the exercises of Fraenkel, which I shall refer to presently. These are directed to the re-education of the comparatively uninjured structures.

I would next allude to diseases of the vascular system causing nervous symptoms. I have already referred to the case of hemiplegia, the result of a rupture of a vessel. When that occurs there is very frequently a tearing across of certain fibres of the nervous system and the destruction of their function. But the destructive effect of a hæmorrhage is usually comparatively small, and the area in which the fibres are completely destroyed is small compared with the area in which the function is interfered with. So in a case of cerebral hæmorrhage one usually finds, in the first instance, a condition of very profound paralysis on one side. I shall not refer to the coma, but to the motor paralysis. If that patient lives, one is justified in saying there will be a considerable degree of recovery, because the congestion and œdema around the area of destruction is very great—sufficient to interfere with the function of a large area of the nervous system. The same is true in a disease like infantile paralysis. Whatever the ultimate pathology of infantile paralysis—anterior poliomyelitis—may be, we know that part of the pathological process certainly is a blocking of blood-vessels and a destruction of cells. But we see almost constantly that the degree of paralysis in the initial stage of the illness is infinitely greater than the ultimate amount of paralysis turns out to be; the patient may at first have both legs paralysed, and the ultimate paralysis may be restricted to one segment of one limb. I know of two boys simultaneously affected with the same kind of illness; they had head retraction and paralysis, and were extremely ill; one recovered perfectly, whereas the other was left with weakness of one leg. I do not doubt for a moment that the boy who completely recovered had anterior poliomyelitis, but in his case the condition cleared up without having effected any serious structural change in his nervous system. Such facts enable us to take a much more hopeful view of the future of such cases: I do not say of treatment, because the treatment of such cases is one of common sense and waiting. We have no drugs of any very great efficacy in those acute diseases.

From the point of view of treatment I wish now to refer to one or two diseases, and first of all, to tabes dorsalis. As you know, it is a disease characterised pathologically by degeneration of the posterior columns of the spinal cord. Clinically it is characterised by the presence of lightning pains, and, in the commonest variety, by what we call hypotonia with ataxy, and not infrequently by some degree of bladder trouble.

In the majority of cases the great difficulty which the patient experiences is in getting about; and Dr. Fraenkel, who was the first to recognise the very close connection between the hypotonia of muscles and the ataxy, devised a series of extremely simple exercises which certainly, if persisted in by the patient, enable him, in many cases, to reacquire the power of walking. The exercises, as illustrated here (various apparatus were shown and the manner of their use in treatment), are simply devised with the view of making the patient practise his co-ordination. The difficulty that the patient has is in moving about freely, unless he keeps his eyes constantly fixed on the ground. In practising these exercises he has to have his eyes directed to the ground, but he is able gradually to get strength in his muscles, so that he is able to get about. Usually he has great difficulty in getting his foot into the footprints, but if he practises very deliberately he is able to do so, though his first difficulty is to be able to balance himself properly on one foot. But give him all the help you can, and if he has extreme ataxy he will need a living support on either side. After he has practised walking more or less deliberately in this way, you can make him perform movements of a more elaborate character; for instance, while resting on one foot make him describe a semicircle before he puts the other foot down, and so also with the other. After he has practised these movements sufficiently, make him walk in the footprints very deliberately, balancing the foot for some time before putting it in the required place. It is almost incredible how much improvement can be effected in some cases by this simple means. The most striking case I have seen was that of a policeman with tabes, who came here some years ago. He had been totally unable to walk for several months, and he had to be carried into the hospital. But in six weeks he went out of the hospital able to walk perfectly well, simply carrying a walking-stick. To bring about such a result both the patient and his nurse must be equally desirous of bringing about the improvement. Of course, in some cases the patient is so ataxic that he is unable to walk or stand at all. I had a patient brought in here about three months ago who was in that condition. He was extremely ataxic, added to the other ordinary symptoms of tabes, and he was so weak and ill that we did not dare to get him out of bed for a week or two after he came in. In such a case we have a device which is put on the bed and which enables the patient to get a certain degree of exercise of the muscles of his legs, and by means of which he can practise co-ordination. The patient is made to practise putting his heels, just over the tendo-achillis, in these slots, one after the other, raising up the foot very deliberately, and putting it into the one which he is told to. It is always well to combine with these exercises the use of massage, because these patients are nearly all badly nourished, and the muscles are considerably weakened, apart from the ataxy. The other apparatus I show you is similar in design, and here we get the patient to put his heel into one or other of these holes. With these before him, a patient, especially an ingenious patient, will devise various exercises, which tend to improve his co-ordination. I think those exercises are of the utmost use in tabes, and I am sure I have seen immense good effected by them, in restoring power which the patients thought they had lost for ever. And that leads me to recall what I spoke of a little while ago, that in some of those cases, apparently, after a certain amount of damage has been done to the nervous system, the poison, or whatever is effecting the changes in the nervous system, ceases to act or ceases to be secreted. I know of one man, an old soldier, who, ten years ago, was living in a bath-chair, but to-day he is acting as librarian in one of the garrison towns; so the fact that a man can improve, who had undoubted tabes of a severe character, is encouraging with reference to other cases.

Besides ordinary cases of ataxy with hypotonia and lightning pains, there are cases of tabes in which the chief symptoms are so-called gastric crises. It is curious that in the majority at least of those cases of gastric crises ataxy is conspicuous by its absence. A patient with gastric crises usually has, at first at least, only the gastric crises, often with loss of knee-jerks and Argyll-Robertson pupils. And the question arises, how are those to be treated? I think a patient with gastric crises has a very bad time indeed, because the vomiting is very severe, and the only thing which, in my experience, controls it is morphia. So a person with gastric crises runs a very great chance of becoming a habitual taker of morphia. I know one patient who passed through the stage of gastric crises without taking morphia, and it is important to recognise that those crises are but a phase of the disease. But to pass through them without taking morphia is a very rare experience. I know another man, who comes here occasionally still, who certainly acquired the morphia habit in a very severe form, and he has recovered from his gastric crises and also from the morphia habit, but he still has absent knee-jerks and Argyll-Robertson pupil. In some cases I have known other trophic changes develop, such as perforating ulcer; and one patient who had a Charcot's joint, went through his phases without having morphia, and developed no other symptom of tabes; and he finally died, after some years, of Bright's disease.

With reference to the type of tabes in which laryngeal crises are a feature, these cases in my experience, are nearly always extremely ataxic. These laryngeal crises depend on the paralysis of the abductors of the vocal cords, and the occurrence of occasional spasm in the adductors. I have never seen a patient die in a laryngeal crisis, but I have seen many patients nearly die from this cause. Usually a patient with such spasm becomes cyanotic, and the spasm passes off. In cases of severity I should not hesitate to use morphia, because that seems to relax the spasm of muscles as quickly as anything I know.

Another point which has to be thought out with reference to the treatment of tabes is in regard to the trophic changes which occur, the so-called perforating ulcers. It is remarkable in what a large number of tabetics the perforating ulcer is caused by the injudicious paring of a corn. Always warn tabetics that they should be extremely careful about their feet, and particularly careful about cutting their nails or cutting corns. These perforating ulcers can often be cured. We used to be taught that when perforating ulcer once appeared it remained for ever. But I have known many cases in which perforating ulcer was cured simply by attention to cleanliness with boracic fomentations and similar treatment. Such a patient has to remain in bed, but the outlook is better than used to be taught.

With regard to the disorganisation of joints in some cases of tabes, associated with Charcot's name, that ought to be treated by support. Wherever there is a Charcot joint there is very great danger of real disaster through the bone perforating the skin. The heads of the bones become absorbed, and the shaft of the bone is sometimes left, and the fact that there is no pain makes the patient take liberties which he would not otherwise take.

There is one sad condition in tabes, namely, optic atrophy. In the vast majority of cases where this occurs, ataxy is conspicuously absent. That is illustrated by a case which I showed on Friday. With reference to treatment of optic atrophy, I am sorry to say that it is a very hopeless problem indeed, although I have known one or two cases in which the atrophy apparently ceased spontaneously. There again it seems as if the supply of poison had been exhausted; either it ceases to be effective, or it is no longer elaborated. At any rate, these patients were left with sufficient vision to enable them to get about. The measures we usually adopt are the hypodermic injection of strychnine, and in some cases it has been claimed that that has led to the cessation of the atrophy. But I am bound to say that, although I have seen cases apparently arrested after considerable damage has been done, I have never seen optic atrophy arrested at a very early stage from any such measure as that. One would also use electricity, either high frequency or galvanic, so as to stimulate the nerve, and get as much out of it as possible; but neither has that, in my experience, been a success.

With regard to disseminated sclerosis, I have already said something about that, but I would add that in many cases the first attack may be the last, and that the repeated attacks, which are so common in the disease, may not occur. And the question arises, what are we to do in such a case so as to favour recovery as much as possible? The routine treatment which I follow here in cases of this character is to put patients to bed, keep them there for three weeks, give them massage, with electrical treatment, so as to improve their nutrition, feed them up as much as possible; and at the end of three weeks I get them up a little, and then more and more as time goes on. In most of the cases this treatment is effective in strengthening them and enabling them to walk better, and apparently in arresting the effect of the attack through which they have just passed. It is particularly good in cases where you get the patient when the attack is still fresh. And you might make the period of rest longer if the attack was very severe. In such cases the tendency is for recovery to take place up to a certain point, but after each attack the patient is left more and more helpless. And what we would like to know is, what determines these attacks. Is it some poison? And if it is, what is the poison? And can we do anything to antagonise it?

There is no special treatment for the optic atrophy which you find in disseminated sclerosis, though it practically never, in that disease, proceeds to complete blindness. There is a patient here whose attack began with complete blindness in both eyes, but that being an inflammatory condition and the result of retro-bulbar neuritis, one expects some improvement, and already he is able to distinguish fingers in front of his eyes, and I anticipate in his case the recovery of a useful amount of vision. I have known patients blind in one eye from the condition. That is contrary to the state of affairs in tabes, because there it is the rule for almost complete blindness to occur.

I will say a few words, in conclusion, with regard to the treatment of epilepsy. Anyone who has much to do with nervous diseases is familiar with epilepsy, and it is a particularly interesting disease, because I do not know any disease in which you can see the effect of drugs so well as in epilepsy. In some cases of epilepsy, unfortunately, you see no effect from drugs; you give all the drugs in the pharmacopœia, more or less, and give various strengths of them, but there is no effect on the fits. These are extraordinary cases, and I always remember one little girl here who had every drug in the Pharmacopœia, and some which are extra-Pharmacopœial. Then we stopped all drugs and sent her to the Convalescent Home at Finchley, and from that day she had no other fit! But in the majority of cases of epilepsy we can do a great deal by means of drugs, and especially by giving attention to dosage, to the time of their administration, and to the combinations of bromides with other drugs. In the ordinary case of epilepsy in which fits occur at long intervals, of course it is often difficult to get a patient to consent to take drugs, but it is always well to start giving 15 or 20 grains of bromide twice or three times a day, and beg him to go on with it for months. And if we find in such a case that the fits increase, we must increase the drugs, or the number of times they are administered in the 24 hours. In that way we have a better means of knowing the patient's co-efficient for the drug, and arriving quickly at the best dosage to suit the case. In cases where the fits occur only at night, one is content to give a fairly large dose of bromide at night. I nearly always give the sodium salt, but occasionally I give strontium, because strontium has a much less tendency to cause acne than has the sodium salt. But in nocturnal epilepsy, I am content to give 20 or even 40 grains of bromide every night, but nearly always combined with digitalis, because, although they are called nocturnal cases, these patients have fits during sleep, and I believe that the fits are occasioned by some change in the circulation which occurs during sleep, and I believe digitalis has a steadying effect on the circulation, and is a very useful adjuvant to bromide in such cases. I also always give bromide with nux vomica, because I am sure that nowadays we do not see, when cases are treated in this way, the depressing effects which used to be so common when bromide of potassium alone was given, and those substances do not interfere with the sedative effect of the bromide. Another drug which one frequently gives in combination is belladonna, and I am sure it is particularly good in children who have attacks of *petit mal*. I have seen cases in children in which bromide and belladonna have stopped attacks when bromide alone had been quite ineffective. Borax is also very useful in combination with bromide, especially in chronic epilepsy. The only drawback to borax is that it is apt to cause gastric disturbance. And—although I have only once seen that—it is apt to cause alopecia. That condition I saw a few weeks ago, in a patient who was taking ten grains three times a day, and he had marked alopecia suddenly developing on the back of his head.

In conclusion, I will only refer to the time of giving the bromide. The ordinary way is to give it three times a day, after each meal, and this is good where the fits are spread over the whole of the day, and when they take place also in the night four doses may be given, though it is often difficult to give the fourth dose. In nocturnal cases I give the medicine at night only. There is a class of case in which the patient has fits in the morning, often during dressing, and in many cases the fits occur only during that time; and in that case you may be certain your treatment will be effective if you give a dose at night, and ask the patient to take another dose half an hour before getting up. In some of those cases, if you stop the fits at one time, they, as it were, crop out at another, and you have to time your doses to deal with the new conditions. But epilepsy is a particularly interesting disease in that way, in that you can draw definite conclusions as to what drugs are doing and what they are not doing. And I think the more one sees of ordinary epilepsy the more one is encouraged to believe that treatment may be effective in controlling fits, or, at all events, so modifying their frequency and severity as to make the patients' condition a much more tolerable one.

